# Interrogating Causal Effects of Body Composition and Puberty‐Related Risk Factors on Adolescent Idiopathic Scoliosis: A Two‐Sample Mendelian Randomization Study

**DOI:** 10.1002/jbm4.10830

**Published:** 2023-10-05

**Authors:** Faegheh Ghanbari, Nao Otomo, Isabel Gamache, Takuro Iwami, Yoshinao Koike, Anas M. Khanshour, Shiro Ikegawa, Carol A. Wise, Chikashi Terao, Despoina Manousaki

**Affiliations:** ^1^ Research Center of the Sainte‐Justine University Hospital University of Montreal Montreal Quebec Canada; ^2^ Laboratory for Statistical and Translational Genetics RIKEN Center for Integrative Medical Sciences, RIKEN Yokohama Japan; ^3^ Department of Orthopedic Surgery Keio University School of Medicine Tokyo Japan; ^4^ Department of Orthopedic Surgery Hokkaido University Graduate School of Medicine Sapporo Japan; ^5^ Scottish Rite for Children Center for Pediatric Bone Biology and Translational Research Dallas Texas USA; ^6^ McDermott Center for Human Growth & Development University of Texas Southwestern Medical Center Dallas Texas USA; ^7^ Department of Pediatrics University of Montreal Montreal Canada; ^8^ Department of Biochemistry and Molecular Medicine University of Montreal Montreal Quebec Canada

**Keywords:** ADOLESCENT IDIOPATHIC SCOLIOSIS, BODY MASS INDEX, BONE MINERAL DENSITY, GENOME‐WIDE ASSOCIATION STUDY, MENDELIAN RANDOMIZATION

## Abstract

Adolescent idiopathic scoliosis (AIS) is the most common form of pediatric musculoskeletal disorder. Observational studies have pointed to several risk factors for AIS, but almost no evidence exists to support their causal association with AIS. Here, we applied Mendelian randomization (MR), known to limit bias from confounding and reverse causation, to investigate causal associations between body composition and puberty‐related exposures and AIS risk in Europeans and Asians. For our two‐sample MR studies, we used single nucleotide polymorphisms (SNPs) associated with body mass index (BMI), waist‐hip ratio, lean mass, childhood obesity, bone mineral density (BMD), 25‐hydroxyvitamin D (25OHD), age at menarche, and pubertal growth in large European genome‐wide association studies (GWAS), and with adult osteoporosis risk and age of menarche in Biobank Japan. We extracted estimates of the aforementioned SNPs on AIS risk from the European or Asian subsets of the largest multiancestry AIS GWAS (*N* = 7956 cases/88,459 controls). The results of our inverse variance‐weighted (IVW) MR estimates suggest no causal association between the aforementioned risk factors and risk of AIS. Pleiotropy‐sensitive MR methods yielded similar results. However, restricting our analysis to European females with AIS, we observed a causal association between estimated BMD and the risk of AIS (IVW odds ratio for AIS = 0.1, 95% confidence interval 0.01 to 0.7, *p* = 0.02 per SD increase in estimated BMD), but this association was no longer significant after adjusting for BMI, body fat mass, and 25OHD and remained significant after adjusting for age at menarche in multivariable MR. In conclusion, we demonstrated a protective causal effect of BMD on AIS risk in females of European ancestry, but this effect was modified by BMI, body fat mass, and 25OHD levels. Future MR studies using larger AIS GWAS are needed to investigate small effects of the aforementioned exposures on AIS. © 2023 The Authors. *JBMR Plus* published by Wiley Periodicals LLC on behalf of American Society for Bone and Mineral Research.

## Graphical abstract

Adolescent idiopathic scoliosis (AIS) is the most common pediatric musculoskeletal disorder. Observational studies have pointed to several risk factors for AIS, but almost no evidence exists to support their causal association with AIS. By applying a Mendelian randomization (MR) study design, we demonstrated a protective causal effect of BMD on AIS risk in females of European ancestry, but this effect was modified by BMI, body fat mass, and 25OHD levels.

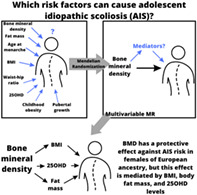

## Introduction

Adolescent idiopathic scoliosis (AIS) is the most common structural spinal deformity, affecting ~3% of the general population in all ethnicities. Approximately 0.3% of children having scoliosis with a spinal curvature of >20° (Cobb angle) require treatment, while more than 1 in 10,000 children develop severe spine deformity requiring surgery.^[^ ^1^ ^]^ The majority of scoliosis cases are considered idiopathic with onset in adolescence, while in rare cases scoliosis occurs as a consequence of disorders such as Marfan syndrome, cerebral palsy, or muscular dystrophy. AIS affects between 1% and 4% of adolescents in the early stages of puberty and is more common in young women than in young men.^[^ ^2^ ^]^ Scoliosis is not always a benign structural abnormality, since severe forms may result in early degenerative joint disease, cardiopulmonary compromise, negative body image, and psychosocial disturbances.^[^ ^3^, ^4^, ^5^ ^]^ Therefore, better understanding of the etiology of scoliosis may provide opportunities for prevention and early intervention.

There are several known risk factors that have been associated with AIS risk in observational studies. For example, inverse associations with risk of AIS have been shown for body mass index (BMI)^[^ ^6^, ^7^ ^]^; aspects of body composition, specifically percentage of fat or lean mass,^[^ ^8^, ^9^ ^]^ fat‐free mass, and predicted muscle mass^[^ ^8^, ^9^ ^]^; bone mass (bone mineral content/density (BMC/BMD)^[^ ^10^ ^]^; 25‐hydroxyvitamin D (25OHD)^[^ ^11^, ^12^ ^]^ and serum calcium^[^ ^13^ ^]^; and leptin levels,^[^ ^8^, ^9^, ^10^ ^]^ while there is a positive association between levels of adiponectin^[^ ^8^ ^]^; and menstrual status and pubertal timing.^[^ ^14^, ^15^ ^]^ However, the problem with the aforementioned epidemiological associations is that they cannot establish causality, since they may be driven by unmeasured confounding or reverse causality. Determining whether the aforementioned risk factors are causal in AIS is important since this would enhance our understanding on the pathophysiology of AIS, while it can inform strategies for primary prevention. Although randomized controlled trials (RCTs) are the gold standard to establish causation, randomization for many of the aforementioned risk factors would be impossible or unethical, while for others (such as vitamin D and calcium levels) only weak evidence from small‐scale RCTs with short follow‐up is available.^[^ ^16^ ^]^

The contribution of genetic factors in the etiology of AIS has been demonstrated by many twin, family and population studies.^[^ ^17^, ^18^ ^]^ Recently, a large trans‐ancestry Genome Wide Association Study (GWAS) on AIS has further unraveled the genetic architecture of AIS.^[^ ^19^ ^]^ Leveraging these GWAS findings, here we used Mendelian randomization (MR), a study design in genetic epidemiology allowing for causal inference, to assess causal associations between risk factors (such as, BMI, waist‐hip ratio, lean mass, childhood obesity, BMD, 25OHD, age at menarche, and pubertal height growth) and AIS risk in both Europeans and Asians.

## Materials and Methods

### MR assumptions

MR uses genetic variants (single‐nucleotide polymorphisms [SNPs]) as instruments to infer levels of an exposure (e.g., a biomarker) and test its effects on an outcome. Since SNPs are allocated randomly at conception (based on Mendel's second law), environmental confounders and disease states occurring later in life cannot affect germline genetic predisposition. As such, MR limits bias due to confounding and reverse causation and allows for causal inference.^[^ ^20^ ^]^ In each MR analysis, three assumptions need to be satisfied: (1) the genetic instrument must be strongly associated with the exposure (relevance assumption); (2) the genetic instrument should not be associated with confounders that link the exposure to the outcome (independence assumption); and (3) the genetic instruments should be associated with the outcome only via the exposure (exclusion restriction assumption). Violation of this assumption is known as horizontal pleiotropy.^[^ ^20^ ^]^ In the next paragraphs, we outline the strategies we undertook to ensure that the foregoing three assumptions are not violated in our MR analyses. We also followed the MR‐STROBE checklist^[^ ^21^ ^]^ to systematically report the methods and results of this study.

### Study exposures

To satisfy the first MR assumption, we used genome‐wide significant and conditionally independent SNPs as instruments for our MR studies. These SNPs were extracted from large available European GWAS for BMI,^[^ ^22^ ^]^ waist‐hip ratio,^[^ ^23^ ^]^ lean mass,^[^ ^24^ ^]^ childhood obesity (defined as a BMI above the 95% percentile in children aged 2 to 18 years),^[^ ^25^ ^]^ BMD,^[^ ^26^ ^]^ 25OHD,^[^ ^27^ ^]^ age at menarche,^[^ ^28^ ^]^ and pubertal growth^[^ ^29^ ^]^ or from GWAS in Biobank Japan (BBJ) for adult osteoporosis risk (see Supplemental materials) and age at menarche^[^ ^30^ ^]^ (Table S1). Since information on BMD is not available in BBJ, adult osteoporosis definition was used as a proxy for BMD in the Japanese population. A recent MR study by our group demonstrated effects of BMI in AIS in Asians,^[^ ^31^ ^]^ while Asian GWAS data for the remaining exposures (waist‐hip ratio, lean mass, childhood obesity, 25OHD, and pubertal growth) are not available, so we could not study their association with AIS in this population.

### Study outcome

To satisfy the second MR assumption and avoid confounding by ancestry in the genetic instruments used in our MR study, we made sure to use data from European‐only or Asian‐only GWAS for both exposures and the outcome. As such, we retrieved the effects of the exposure‐associated SNPs on AIS in the European or Asian subset of the largest AIS GWAS available to date.^[^ ^19^ ^]^ The European GWAS subset contains a meta‐analysis of four non‐Hispanic white cohorts (Texas‐GWAS1,^[^ ^32^ ^]^ Texas‐GWAS2,^[^ ^33^ ^]^ Texas‐GWAS3, and Missourri‐MO1) totaling 1503 cases and 18,594 controls.^[^ ^19^ ^]^ The Asian GWAS subset contains 5327 cases and 73,884 controls from BBJ. The definition of AIS in all GWAS cohorts was based on a Cobb angle above 10°. The average Cobb angle was calculated in one subcohort of the European AIS (Texas‐GWAS1) as detailed in Sharma et al.^[^ ^32^ ^]^ In summary, the Cobb angle of the major spinal curve at ascertainment was used. Cases that had been surgically corrected at the time of ascertainment were not included in the average calculation. Given that AIS is overrepresented in females, we sought to increase our yield to detect associations by restricting our MR analyses to the female subset on the AIS GWAS for both ethnicities. We also restricted our MR analyses testing as exposure age at menarche to European and Asian females, using the female subsets of the respective AIS GWAS. Therefore, we retrieved the effects of the aforementioned SNP instruments from the female subset of the European AIS GWAS, totaling 1278 cases and 10,630 controls, and from the female subset of the Asian GWAS, totaling 5004 cases and 33,679 controls. The AIS cases (in the entire cohort) had an average age of 14.6 years (range of 12.2 to 17 years), an average BMI of 20.8 kg/m^2^ (ranging from 18.6 to 23 kg/m^2^), and an average Cobb angle of 33.1° (ranging from 21.4° to 44.5°).^[^ ^32^ ^]^ In the Japanese cohort, the AIS cases had an average age of 14.9 years (range of 10 to 18 years), an average BMI of 19.1 kg/m^2^ (range of 13 to 26 kg/m^2^), and an average Cobb angle of 37.7°(ranging from 11° to 130°). All controls in both European and Japanese subsets of the GWAS had no musculoskeletal or neurological disorders.

### Statistical analyzes

#### MR analyses

To test causal associations between the exposures (BMI, waist‐hip ratio, lean mass, childhood obesity, BMD, 25OHD, age at menarche, and pubertal growth) and AIS in our two‐sample MR analyses, we first extracted genome‐wide significant SNPs for our eight exposures. For the European MR analyses, all SNP instruments had a GWAS *p* value for association with the exposures of <5 × 10^−8^. For the Asian MR analyses, studying the association of adult osteoporosis and age at menarche with the outcome, the SNP instruments had a genome‐wide suggestive *p* value of <5 × 10^−6^, since very few instruments achieved a genome‐wide significant *p* value in the Asian AIS GWAS. For SNPs not present in the European AIS GWAS, the SNPs in high linkage disequilibrium (LD) (defined by an LD *R* ^*2*^ ≥ 0.7 in the 1000 Genomes phase 3 European panel) were selected as proxies using the LD proxy function in ldlink (https://ldlink.nci.nih.gov/?tab=ldproxy). We next performed LD clumping as implemented in the TwoSampleMR package^[^ ^20^ ^]^ to ensure that the genome‐wide significant SNPs for each exposure were not in LD (*r* ^2^ < 0.001). As a further step to assess the first MR assumption, we estimated the strength of our genetic instrument by computing the F‐statistic metric (with a *F*‐statistic >10 implying a strong instrument) using the following formula: *F* = (*R* ^2^/*k*)/([1 − *R* ^2^]/[*n* − *k* − 1]), where *R* ^2^ is the proportion of the variance of each exposure explained by the SNP instruments, *k* is the number of instruments used in the model, and *N* is the exposure GWAS sample size.^[^ ^34^ ^]^ The variance explained of each exposure by its respective genetic instruments (*R* ^2^) was the addition of the variance explained by each SNP, which we calculated using the following formula: *R* ^2^ ≈ 2β^2^ƒ(1– ƒ), where β and ƒ denote respectively the effect estimate and the effect allele frequency of the allele.^[^ ^35^ ^]^

We then computed the MR Wald ratios for each genetic instrument of the exposures and meta‐analyzed them using the inverse variance‐weighted (IVW) method. To ensure that the third MR assumption was not violated,^[^ ^20^ ^]^ we applied different approaches to account for potential horizontal pleiotropy. First, we verified the heterogeneity of the SNP instruments (using the Cochran Q metric) for each exposure and generated MR estimates omitting SNPs appearing as outliers^[^ ^36^ ^]^ using the Mendelian randomization pleiotropy RESidual Sun and Outlier (MR‐PRESSO) method.^[^ ^36^ ^]^ To account for potential unmeasured pleiotropy, we applied MR‐Egger regression,^[^ ^37^ ^]^ which estimates an intercept as a measure of the average pleiotropic effect and generates a slope coefficient as a MR estimate robust to pleiotropy. MR‐Egger requires the association of each variant with an exposure not be correlated with its pleiotropic effect (known as the InSIDE assumption), in order to weaken the exclusion restriction assumption.

In addition, a weighted median analysis was performed^[^ ^38^ ^]^ to weight individual MR estimates by their precision. This method is based on the fact that SNP estimates without pleiotropic effects tend to merge toward the median; however, pleiotropy may introduce heterogeneity and relative outliers. When less than 50% of the total weight derives from variants with pleiotropic effects, a applying weighted median approach provides reliable results. Finally, we used the weighted mode approach, which is similar to the weighted‐median approach; however, it uses a mode‐based estimate rather than the median‐based one. This approach allows for the majority of SNPs to be pleiotropic.^[^ ^39^ ^]^

All the aforementioned methods were applied as sensitivity analyses since using different pleiotropic assumptions ensures that there will be a lower probability that our findings will be biased by pleiotropy. In addition, we opted to use random‐effects IVW MR when heterogeneity was identified by the Cochran Q metric. To compute the four different MR estimates (IVW, MR‐Egger, weighted median, and weighted‐mode) for the main analysis, we used the TwoSampleMR R package^[^ ^40^ ^]^ and its default parameters. The same R package was used to generate scatter plots to illustrate the estimates using different MR methods. To further control for pleiotropy, we applied the global test, outlier test, and distortion test using the MR pleiotropy residual sum and outlier (MR‐PRESSO) R package.^[^ ^36^ ^]^ Moreover, whenever we detected a causal effect of an exposure on AIS, we undertook multivariable MR to explore effect modification after adjusting for other exposures representing potential confounders or mediators using the multivariable MR (MVMR) package.^[^ ^41^ ^]^ A variable can serve as a mediator when it lies along the pathway between an exposure and an outcome. On the other hand, a confounder affects both the exposure and the outcome, potentially introducing bias if not properly addressed. Finally, since in the two Asian MR analyses there was a partial overlap of samples between the exposure and outcome GWAS, we accounted for bias due to this by recalculating the IVW estimates using the MRlap method,^[^ ^42^ ^]^ which also computes the statistical difference between unadjusted and adjusted (for sample overlap) IVW estimates.

#### Statistical power analysis

To test whether our study was adequately powered to detect clinically relevant changes in AIS risk, we used a previously described MR power calculation method.^[^ ^43^ ^]^ Specifically, we calculated the MR odds ratio (OR) for each exposure for which we obtained a power of 80%, setting the alpha level at 0.05, using the variance explained of each exposure by its respective genetic instruments, and a sample size of the European AIS cohort of 20,096 (among which 1503 cases)^[^ ^19^ ^]^ and of the Japanese AIS cohort of 79,211 (among which 5327 cases). We repeated the power analysis for the female‐only MR studies in both Europeans (1278 cases and 10,360 controls) and in Japanese (5004 cases and 33,679 controls).

## Results

### Main MR analyses

**Fig. 1:**
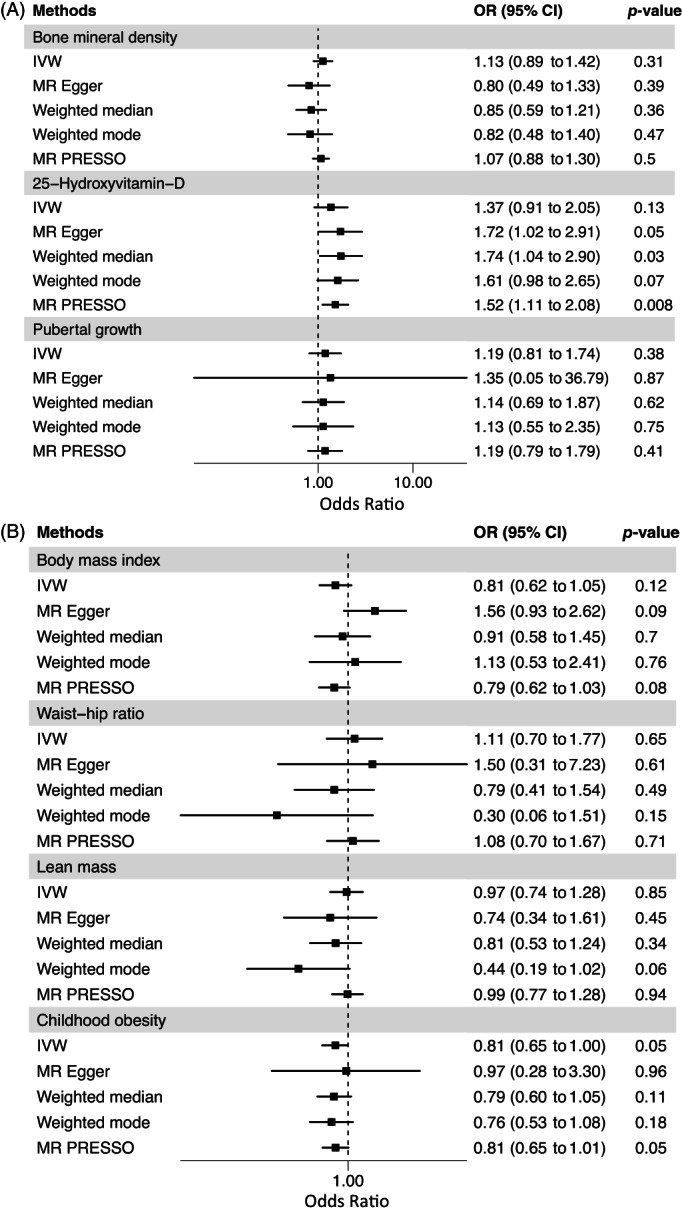
Forest plot of MR study investigating effects of clinical risk factors on risk of adolescent idiopathic scoliosis (AIS) in Europeans. Forest plot depicting effect of (A) BMD, 25OHD, age at menarche and pubertal growth; (B) BMI, waist‐hip ratio, lean mass, and childhood obesity on AIS in Europeans. 25OHD = 25‐hydroxyvitamin D; BMD = bone mineral density; BMI = body mass index; IVW = inverse variance‐weighted method.

As shown in Table 1, we did not find evidence supporting a causal association between the exposures (BMI, waist‐hip ratio, lean mass, childhood obesity, BMD, 25OHD, and pubertal growth)^[^ ^22^, ^23^, ^24^, ^25^, ^26^, ^27^, ^28^, ^29^ ^]^ and risk of AIS in Europeans.^[^ ^19^ ^]^ For instance, we observed nonsignificant IVW MR estimates on AIS for waist‐hip ratio (IVW MR OR = 1.11, 95% confidence interval [CI]: 0.70 to 1.77, *p* = 0.65 per SD increase in waist‐hip ratio), lean mass (IVW MR OR = 0.97, 95% CI: 0.74 to 1.28, *p* = 0.85 per SD increase in mass of fat‐free arms and legs), BMD (IVW MR OR = 1.13, 95% CI: 0.89 to 1.42, *p* = 0.31 per SD increase in estimated BMD), 25OHD (IVW MR OR = 1.37, 95% CI: 0.91 to 2.05, *p* = 0.13 per SD increase in naturally log‐transformed 25OHD level), pubertal growth (IVW MR OR = 1.19, 95% CI: 0.81 to 1.74, *p* = 0.38 per SD increase in modeled pubertal height growth spurt), BMI (IVW MR OR = 0.81, 95% CI: 0.62 to 1.05, *p* = 0.12 per SD increase in BMI), and childhood obesity (IVW MR OR = 0.81, 95% CI: 0.65 to 1.00, *p* = 0.05 for genetic liability to childhood obesity). The same results were confirmed using the other three pleiotropy‐robust MR methods (Fig. 1). Moreover, there was no evidence of heterogeneity in the MR instruments for all the exposures, except for waist‐hip ratio (Cochran's Q *p* value = 0.02). Therefore, in our MR examining effect of the waist‐to‐hip ratio on AIS, we performed random‐effects IVW MR. However, for this MR study the intercept of the MR‐Egger regression was not significant (*p* value intercept = 0.77), as was the MR‐PRESSO global test (*p* value global test = 0.71), implying an absence of horizontal pleiotropy (Table S2).

**Table 1:** Results of Inverse Variance‐Weighted MR for Exposures and Risk of Adolescent Idiopathic Scoliosis

Exposure	Odds ratio	95% CI	*p*	No. SNPs	*R* ^2^	*F*‐ statistic	MR egger intercept‐*p* value	Cochran's Q‐*p* value
Childhood obesity	0.81	0.65 to 1.00	0.05	8	8.77	261.54	0.79	0.74
Bone mineral density (Europeans)	1.13	0.89 to 1.42	0.31	299	8.66	112.82	0.04	0.05
Adult osteoporosis risk (Japanese)	0.97	0.84 to 1.09	0.6	19	4.58	195.24	0.23	0.1
Lean mass	0.97	0.74 to 1.28	0.85	297	7	98.58	0.07	0.08
Body mass index	0.81	0.62 to 1.05	0.12	478	6.2	85.18	0.02	0.07
Pubertal growth	1.19	0.81 to 1.74	0.38	7	2.87	74.79	0.94	0.38
Waist‐hip ratio	1.11	0.7 to 1.77	0.65	217	2.26	71.12	0.77	0.02
Waist‐hip ratio (random effects)	1.08	0.7 to 1.7	0.71	217	2.26	71.12	0.77	0.01
25OHD	1.37	0.91 to 2.05	0.13	46	2.43	228.53	0.15	0.67

*Note*: Analyses were performed in Europeans unless otherwise specified. All IVW analyses are fixed effects unless otherwise specified.

Abbreviations: CI = confidence interval; IVW = inverse‐variance weighted; MR = Mendelian randomization; *R* ^2^ = variance explained; SNP = single‐nucleotide polymorphism.

Our MR analyses showed no causal association between genetic liability to adult osteoporosis and the risk of AIS in the Asian population (IVW MR OR = 0.97, 95% CI: 0.84–1.09, *p* = 0.78 per SD increase in BMD), and results were consistent using all sensitivity MR methods, including the MRlaps method accounting for sample overlap (Tables 1, S2, S3 and Fig. S2).

### Female‐only MR analyses

**Fig. 2:**
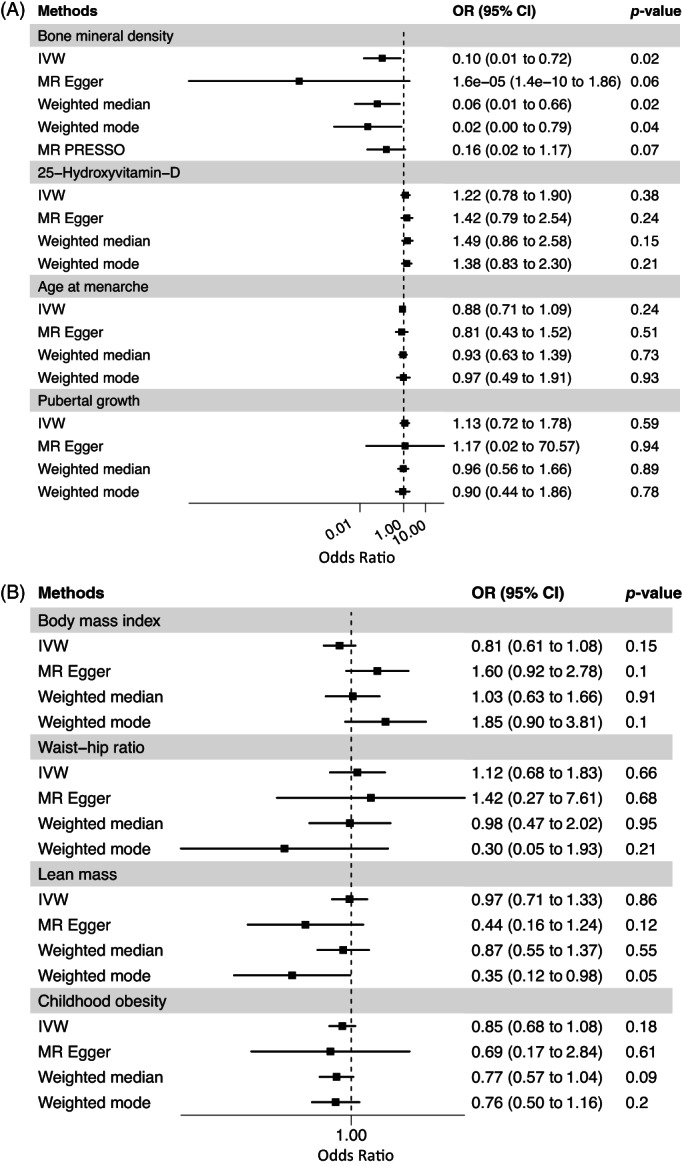
Forest plot of MR study investigating effects of clinical risk factors on risk of adolescent idiopathic scoliosis (AIS) in European females. Forest plot depicting effect of (A) BMD, 25OHD, age at menarche and pubertal growth; (B) BMI, waist‐hip ratio, lean mass, and childhood obesity on AIS in European females. MR PRESSO method was only used for BMD as exposure. 25OHD = 25‐hyrdoxyvitamin D; BMD = bone mineral density; BMI = body mass index; IVW = inverse variance‐weighted method.

**Fig. 3:**
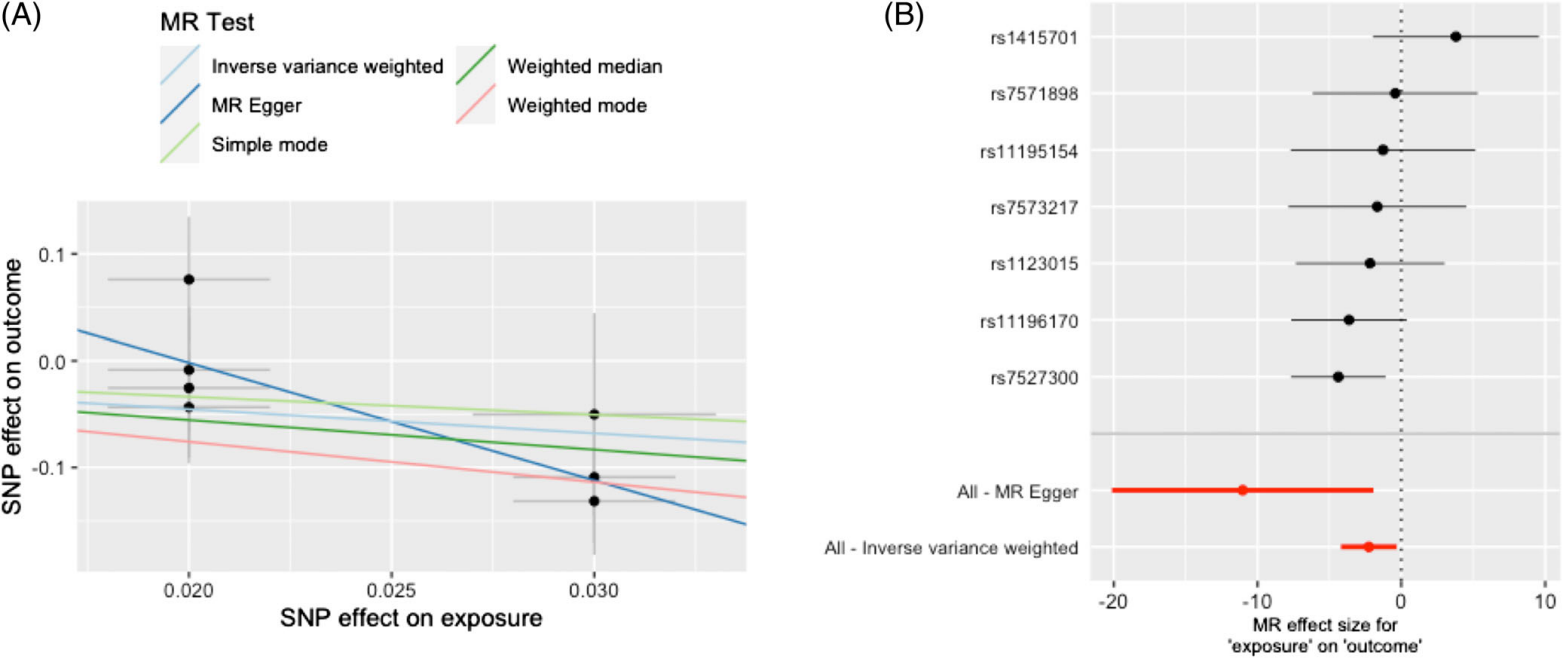
Scatter plot and forest plot of MR study investigating effects of bone mineral density (BMD) on risk of adolescent idiopathic scoliosis (AIS) in European females. (A) Scatter plot of BMD on AIS; *x*‐axis represents genetic association with BMD, *y*‐axis represents genetic association with risk of AIS. (B) Forest plot of MR study of effect of BMD on risk of AIS per SD increase in estimated BMD.

We repeated the MR analyses for the aforementioned exposures using the female subset of European AIS GWAS. Our results revealed a causal association between BMD and AIS risk (IVW MR OR = 0.10, 95%CI 0.01 to 0.72, *p* = 0.02 per SD increase in estimated BMD) (Table 2, Figs. 2A and 3). This means that the risk of AIS decreases by approximately 90% per SD increase in estimated BMD in females of European ancestry. Moreover, as shown in Table 2, we found no evidence of heterogeneity among the SNP instruments for BMD. However, we observed that the intercept of the MR‐Egger regression was significant (*p* = 0.037) (Table 2), suggesting the presence of unbalanced horizontal pleiotropy, which was not supported by MR‐PRESSO (*p* value global test = 0.37) (Table S2). As shown in Fig. 2A, the MR results from weighted median and weighted mode methods also supported the causal association between BMD and AIS in females, while the result of MR‐Egger was suggestive (MR Egger OR = 1.62 × 10^−5^, 95% CI: 1.4 × 10^−10^ to 1.86, *p* = 0.06). The remaining exposures (BMI, waist‐hip ratio, lean mass, childhood obesity, 25OHD, age at menarche, and pubertal growth) were not causally associated with female AIS in our MR analyses (Figs. 2A,B, S1, Table 2). Our Asian MR results in females only did not support an association between genetic liability to adult osteoporosis and age at menarche and the risk of AIS, and this result was consistent using different MR methods (Table 2, Fig. S2).

**Table 2:** Results of Inverse Variance‐Weighted MR for Exposures and Risk of Adolescent Idiopathic Scoliosis in Females

Exposure	Odds ratio	95% CI	*p*	No. SNPs	MR egger intercept‐*p* value	Cochran's Q‐*p* value
Age at menarche (Europeans)	0.88	0.71 to 1.09	0.24	172	0.84	0.81
Age at menarche (Japanese)	1.02	0.84 to 1.19	0.8	41	0.84	0.09
Childhood obesity	0.85	0.68 to 1.08	0.18	8	0.76	0.71
Bone mineral density (Europeans)	0.10	0.01 to 0.72	0.02	7	0.037	0.05
Adult osteoporosis risk (Japanese)	0.99	0.77 to 1.2	0.85	14	0.48	0.67
Lean mass	0.97	0.71 to 1.33	0.87	238	0.03	0.12
Body mass index	0.81	0.61 to 1.08	0.15	478	0.02	0.21
Pubertal growth	1.13	0.72 to 1.78	0.6	7	0.98	0.28
Waist‐hip ratio	1.12	0.68 to 1.83	0.67	217	0.88	0.08
25OHD	1.22	0.78 to 1.90	0.39	46	0.84	0.78

*Note*: Results refer to Europeans unless otherwise specified. All IVW analyses are fixed effects unless otherwise specified.

Abbreviations: CI = confidence interval; IVW = inverse variance‐weighted; MR = Mendelian randomization; *R* ^2^ = variance explained; SNP = single‐nucleotide polymorphism.

Based on recent data from our group in Asians,^[^ ^31^ ^]^ BMI is genetically correlated to AIS risk in this ethnic group. Given the known association between BMI and BMD^[^ ^44^, ^45^ ^]^ we sought to verify whether the causal effect of BMD on female AIS in our MR study was independent of the effect of BMI. To test this, we performed MVMR. The effect of BMD on female AIS risk disappeared after adjusting for BMI (IVW MR OR = 0.18, 95% CI: 0.01 to 3.31, *p* = 0.25 per SD increase in estimated BMD, Table S4), suggesting that the causal effect of BMD on the risk of AIS was dependent on BMI, either by mediation or by a confounding effect. We used the same MVMR approach to test whether the causal effect of BMD on AIS in female AIS is independent of the other risk factors, such as body fat mass, 25OHD, and age at menarche. As shown in Table S4, our results demonstrated that the causal effect of BMD on AIS was not significant after adjusting for body fat mass (IVW MR OR = 0.16, 95% CI: 0.02 to 1.33, *p* = 0.09 per SD increase in estimated BMD), and 25OHD (IVW MR OR = 0.2, 95% CI: 0.03 to 1.45, *p* = 0.11 per SD increase in estimated BMD), but remained significant after adjusting for age at menarche (IVW MR OR = 0.11, 95% CI: 0.01 to 0.81, *p* = 0.03 per SD increase in estimated BMD).

### MR power analysis

Based on a sample size of 20,096 individuals (among which were 1503 cases of both sexes) and setting alpha to 0.05, our MR study had 80% power to detect effects on AIS as small as an OR ranging from 1.26 to 1.5 per SD change of the different exposures in Europeans. The results of the power analysis in the Japanese cohort showed minimal ORs for AIS of 1.17 and 1.30 for BMD and age at menarche as exposures, respectively. The minimal ORs for our MR analyses in females only were slightly higher (Table S5).

## Discussion

Leveraging data from the largest available GWAS consortia for AIS and the studied exposures, our MR studies did not find evidence for a causal association between BMI, waist‐hip ratio, lean mass, childhood obesity, BMD, 25OHD, age at menarche, and pubertal growth and the AIS risk in Europeans or between risk of adult osteoporosis or age at menarche and AIS risk in Asians. Our findings were consistent using different MR methods sensitive to pleiotropy. However, when we restricted our MR analyses to European females with AIS, we detected a causal association between genetically determined change in the estimated BMD and risk of AIS, which is conditional on BMI, body fat mass, and 25OHD. It is important to mention that our MR studies can confidently exclude effects larger than an OR of 1.17 to 1.5, and therefore smaller effects cannot be excluded, and future well‐powered MR studies are needed to investigate such effects.

We are among the first to assess causal associations of body composition and puberty‐related exposures with AIS risk using MR. Our MR finding supporting a causal association between BMD and female AIS risk is in line with several observational studies that reported a higher prevalence of low BMD among women with AIS.^[^ ^46^, ^47^, ^48^, ^49^ ^]^ For instance, Hung et al.^[^ ^50^ ^]^ showed that, in a cohort of 324 girls with AIS followed up until skeletal maturity or progression of curves ≥6°, low BMD was a significant prognostic factor for curve progression. Moreover, a double‐blinded RCT also demonstrated that using calcium and vitamin D supplementation could improve bone strength and prevent curve progression in adolescent girls with AIS.^[^ ^16^ ^]^ Interestingly, our MVMR findings demonstrate that the causal association of BMD with female AIS is dependent on the effects of BMI, body fat mass, and 25OHD. These risk factors could potentially function as either mediators or confounders, given that epidemiological associations have been described between both scoliosis and BMD and BMI,^[^ ^6^, ^7^, ^51^, ^52^, ^53^, ^54^, ^55^, ^56^ ^]^ body fat mass,^[^ ^9^, ^53^, ^54^, ^55^, ^56^, ^57^ ^]^ and 25OHD.^[^ ^13^, ^58^, ^59^ ^]^ Regarding BMI and body fat mass, the hormone leptin, which is secreted by fat cells, has a negative impact on bone mass^[^ ^60^ ^]^ and a positive impact on the muscle mass,^[^ ^9^ ^]^ suggesting that being underweight could impair postural balance, favoring a risk of developing scoliosis. Despite this, we did not observe a causal association between BMI and AIS risk in our MR analyses in Europeans (in both sexes or in females only), but a recent study from our group showed a genetic correlation between BMI and AIS in Asians.^[^ ^61^ ^]^ In addition, it has been reported that adipose tissue can indirectly affect bone metabolism via adipokine, cytokines, and hormones and can stimulate bone formation by increasing circulating leptin levels.^[^ ^62^, ^63^ ^]^ The fact that we were able to detect an association between BMD and AIS risk only in European females but not in Asian females and in the entire AIS cohort, comprising both males and females, is interesting and suggests possible distinct pathophysiological pathways across sexes and ethnicities.

With regard to a confounding or mediating role of 25OHD in the BMD–AIS association, evidence from observational studies has linked low BMD in AIS patients with vitamin D deficiency and, interestingly, latency at the age of menarche.^[^ ^58^, ^64^, ^65^, ^66^ ^]^ Also, several studies reported a positive correlation between 25OHD level and BMD in children and adolescents,^[^ ^58^ ^]^ other studies supported a link between early menarche with higher peak bone mass.^[^ ^67^ ^]^ Possible mechanisms include the role of vitamin D in facilitating calcium absorption and bone mineralization, which in turn can affect BMD.^[^ ^68^ ^]^ Vitamin D may also have an effect on the central nervous system, impacting postural balance, and on estrogen production, both of which can influence the risk of developing scoliosis.^[^ ^11^ ^]^ Although our MR findings did not reveal a causal association between 25OHD and age at menarche and AIS risk, the causal MR effect of BMD on female AIS risk may depend on 25OHD levels, but not on age at menarche. Our remaining null MR findings are in contrast to several observational studies that reported a positive association between adult and pediatric BMI, waist‐to‐hip ratio, 25OHD, age at menarche, and pubertal growth and the risk of AIS.^[^ ^6^, ^7^, ^8^, ^9^, ^10^, ^11^, ^12^ ^]^ However, these observational studies may suffer from confounding and reverse causation.

Our MR approach has several strengths. First, we used data from the European subset of the largest multiethnic AIS GWAS available to date^[^ ^19^ ^]^ and the largest GWAS available for the studied exposures.^[^ ^22^, ^23^, ^24^, ^25^, ^26^, ^27^, ^28^, ^29^ ^]^ Second, our MR approach allows for causal inference by limiting the bias from confounding or reverse causation that are present in observational studies. Reverse causation is of particular importance when studying the association of epidemiological risk factors and AIS. For instance, limitation in physical activity in individuals with severe AIS can lead to low BMD and low 25OHD levels. Finally, by applying a two‐sample MR approach, we maximized our power to detect associations between the exposures (measured in large European GWAS) and AIS.

Our analysis also has some limitations worth considering. The small number of cases in our European AIS GWAS limited our power to detect very small effects (OR <1.17–1.5). We were still well powered to exclude effects that were small enough to be clinically relevant, based on what was previously reported in observational studies with ORs ranging from 0.8 to 2.2.^[^ ^6^, ^7^, ^8^, ^9^, ^10^, ^11^, ^12^, ^14^, ^15^ ^]^ Future MR studies, using larger European AIS GWAS, are needed to validate our findings. In addition, we did not use SNPs from female‐only GWAS for exposures, such as BMD, BMI, body fat mass, and 25OHD in our female‐only AIS MR. Nevertheless, the effects of SNPs are adjusted for sex in the aforementioned GWAS, and, with a few exceptions, genetics of biomarkers are shared between males and females^[^ ^69^ ^]^; therefore, we do not think that this significantly affected the results of our analysis.

Also, for some exposures, such as the BMD, GWAS were performed in adults of a more advanced age than the age of participants in the AIS GWAS, and one could argue that genetic variants affecting these exposures could be age‐dependent. However, genetic variants affecting traits measured in childhood and in adulthood largely overlap, and this has been shown for BMD.^[^ ^70^, ^71^ ^]^ In the Asian MR analyses, GWAS data for both the exposures (osteoporosis risk, age at menarche) and the outcome (AIS) were from BBJ, and therefore these analyses were equivalent to one‐sample MR studies. While the risk of type 1 error (false positive findings) is increased in the one‐sample MR setting,^[^ ^72^ ^]^ given our null results in our Asian MR analyses, this is less of a concern here. Furthermore, there is sample overlap between the GWAS for scoliosis and the two outcomes (osteoporosis and age at menarche) in the Asian MR analyses, which can cause bias. Nevertheless, using the MRlap method,^[^ ^42^ ^]^ which adjusts for sample overlap, we obtained results similar to those in the main MR analysis, which eliminates the presence of a strong bias. Also, the fact that only controls for AIS overlapped with samples in the osteoporosis and age at menarche GWAS also reduced the impact of potential bias due to sample overlap in the Asian MR analyses.^73^

The two‐sample MR design of our European MR studies did not allow for the assessment of nonlinear effects, and such effects were not tested in stratified MR in BBJ; thus, we cannot exclude the possibility that an individual at the extremes of the normal distribution of the different exposures could have an altered risk of AIS. Moreover, although GWAS for both MR exposures and outcome (AIS) were restricted to either Europeans or Asians and adjusted for ancestral principal components in both populations, residual population stratification could violate the second MR assumption.^[^ ^74^ ^]^ In the analysis using the Asian dataset, due to the limited number of genome‐wide significant SNPs, MR analyses were performed with a relaxed threshold to select instrumental variables. This could increase the risk of weak instrument bias. Also, aside from commonly utilized covariates like age and sex, the Asian GWAS for osteoporosis integrated additional heritable covariates, such as type 2 diabetes, obstructive pulmonary disease, and chronic kidney disease. This could induce bias in the MR analysis in case of presence of residual confounding between the aforementioned covariates and osteoporosis, even in the absence of horizontal pleiotropy.^[^ ^75^ ^]^ As such, the results of this analysis should be interpreted with caution. Finally, our MR results cannot be generalized to ethnicities other than those studied.

In conclusion, our MR findings reveal a protective causal association between BMD and female AIS in Europeans, but this effect is conditional on BMI, body fat mass, and 25OHD levels. Moreover, our findings did not support a causal association between BMI, waist‐hip ratio, lean mass, childhood obesity, 25OHD, age at menarche, and pubertal growth and the risk of AIS in Europeans and between BMD and age of menarche and AIS in Asians, but small effects cannot be excluded. The findings reported here enhance our understanding of the pathophysiology of AIS and can inform preventive strategies.

## Author Contributions

**Faegheh Ghanbari:** Data curation; formal analysis; writing – original draft; writing – review and editing. **Nao Otomo:** Data curation; writing – original draft. **Isabel Gamache:** Data curation. **Takuro Iwami:** Data curation. **Yoshinao Koike:** Data curration. **Shiro Ikegawa:** Conceptualization; supervision. **Carol A. Wise:** Conceptualization. **Chikashi Terao:** Conceptualization; supervision. **Despoina Manousaki:** Conceptualization; formal analysis; funding acquisition; methodology; project administration; resources; supervision; writing – original draft; writing – review and editing.

## Disclosures

No potential conflicts of interest relevant to this article were reported.

### Peer Review

The peer review history for this article is available at https://www.webofscience.com/api/gateway/wos/peer-review/10.1002/jbm4.10830.

## Supporting information


**Data S1.** Supporting Information.

## Data Availability

R scripts used to generate the results of this study are available upon request to the corresponding author. Summary‐level results of all GWAS used in this study are publicly available through the GWAS catalog, except from the Japanese adult osteoporosis GWAS, for which data are available upon request from the corresponding author.
