# Supplementary material for: Interrogating Causal Effects of Body Composition and Puberty‐Related Risk Factors on Adolescent Idiopathic Scoliosis: A Two‐Sample Mendelian Randomization Study
Source: JBMR Plus. 2023 Oct 5;7(12):e10830. doi: 10.1002/jbm4.10830 (PMC10731118; doi:10.1002/jbm4.10830)
Supplement: Supplementary file 1 — Data S1. Supporting Information. [file JBM4-7-e10830-s001.docx]

**Supplemental materials**

**Supplemental Methods**

**Adult osteoporosis GWAS in Biobank Japan**

**Phenotype definition**: In the main GWAS analysis, we analyzed 7, 246 cases with adult osteoporosis, and 71,788 controls from BioBank Japan (BBJ) ^(1, 2)^ . Additionally, a sex-specific GWAS was performed, including only women (6,177 cases/30,688 controls). The BBJ is a multi-institutional hospital-based registry that collected DNA, serum, and clinical information of approximately 200,000 patients from 66 hospitals affiliated with 12 medical institutes between fiscal years 2003 and 2007. All study participants had been diagnosed with one or more of 47 target diseases by physicians at the cooperating hospitals. Adult osteoporosis was defined based on the following criteria: 1. Osteoporosis diagnosed by a physician 2. Over 50 years of age subjects with a history of vertebral compression fracture 3. Over 50 years of age subjects with a history of femoral neck fracture. We excluded patients with diseases that secondarily affect bone metabolism (e.g., collagen disease, cancer, thyroid disease, and Cushing’s syndrome) and patients likely to be using drugs that secondarily affect bone metabolism (e.g., steroids, warfarin, and antiepileptic drugs). Controls were subjects over 50 years of age without the 3 above osteoporosis definition criteria.

**Genotyping, imputation and association study:** Genotyping of the BBJ was performed using the Illumina OmniExpressExome BeadChip or a combination of the Illumina OmniExpress BeadChip and the Illumina HumanExome BeadChip. As part of the quality control, we excluded SNPs with call rate < 99%, minor allele count > 5, and Hardy-Weinberg equilibrium P*-*value < 1.00 × 10^-6^. We calculated the estimates of pairwise IBD (PI_HAT) and removed monozygotic twins and duplicate samples that showed PI_HAT > 0.9. We estimated the population stratification using principal component analysis (PCA) with four populations from HapMap data as the reference: European (CEU), African (YRI), Japanese (JPT), and Han Chinese (CHB) using SmartPCA ^(3).^ We excluded samples outside the East Asian (JPT/CHB) cluster. We excluded samples with a genotyping call rate of < 98%. Following quality control, we retained 79,034 individuals with BMD phenotype is our main GWAS, and we also performed a female-only BMD GWAS was performed, in 36,865 women.

Then, we constructed a reference panel for imputation using the 1000 Genomes Project Phase 3 [1KGP 3 (May 2013, n = 2,504)] and 3,256 in-house Japanese whole-genome sequence data obtained from BBJ in the same way as previously reported ^(4)^. Phasing using EAGLE (v2.4.1) ^(5)^ and imputation using minimac4 (v1.0.0) ^(6)^ were performed. For the association study, we used SAIGE ^(7)^, adjusting for age, gender, PC1-5, chronic kidney disease, chronic obstructive pulmonary disease, and type 2 diabetes mellitus as covariates.

**Supplemental Figures**

**Supplementary Figure S1. Scatter plots of the MR studies demonstrating the effects of clinical risk factors (A-H) on risk of Adolescent Idiopathic Scoliosis (AIS) in Europeans. A)** Body Mass Index, **B)** Waist-Hip ratio, **C)** Lean Mass, **D)** Childhood Obesity, **E)** Bone Mineral Density, **F)** 25-hydroxyvitamin D, **G)** Age at menarche (female AIS), **H)** Pubertal Growth.


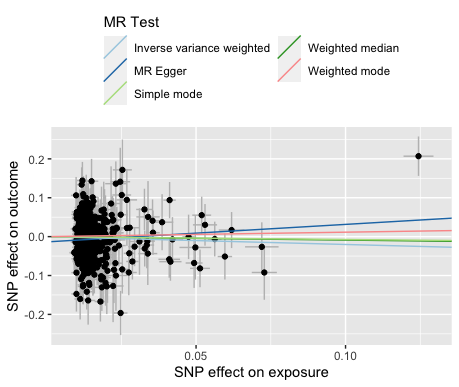

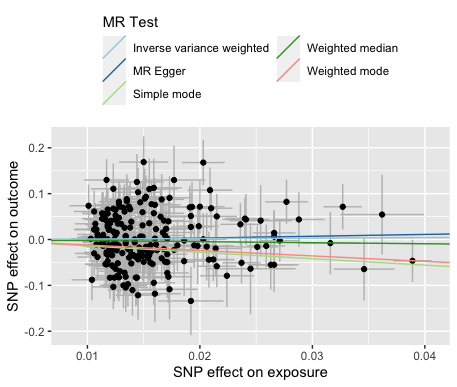


**B**

**A**

**
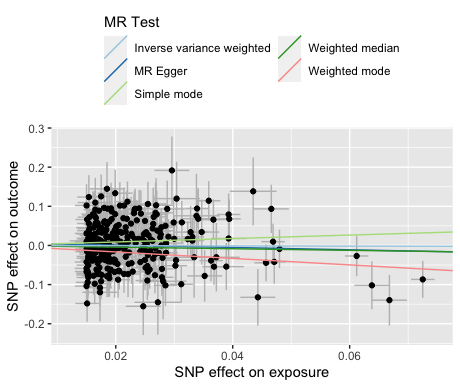

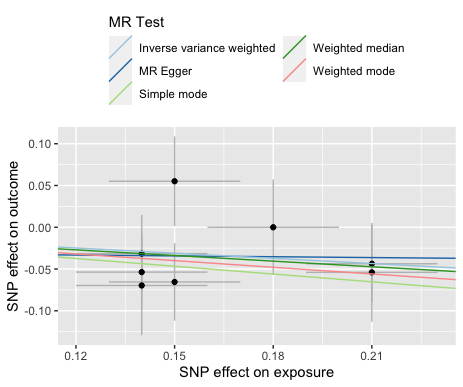
**

**D**

**C**

**
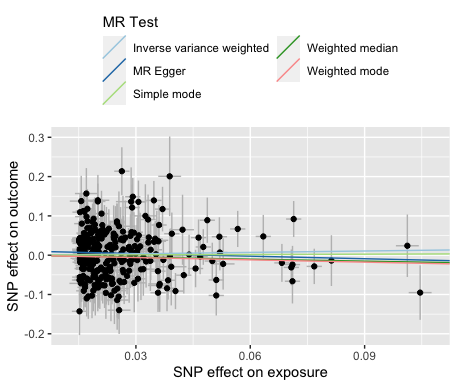

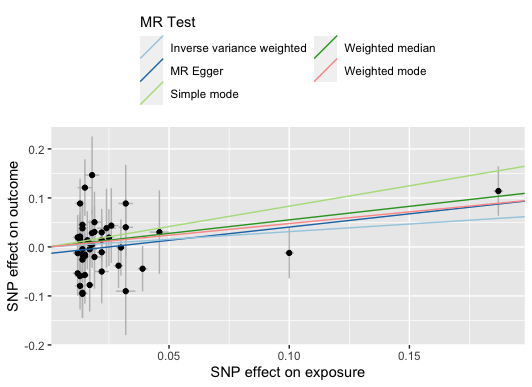
**

**F**

**E**

**
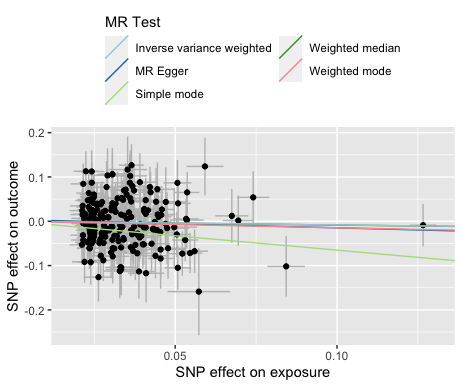

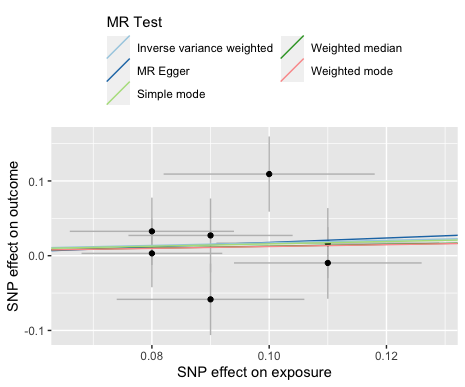
**

**H**

**G**

**Supplementary Figure S2.** Scatter plots of the MR studies demonstrating the effects of clinical risk factors (A,B,C) on risk of Adolescent Idiopathic Scoliosis (AIS) in Asians. **A)** Genetic liability to osteoporosis (both sexes), **B)** Genetic liability to osteoporosis (female AIS), **C)** Age at menarche (female AIS).

**
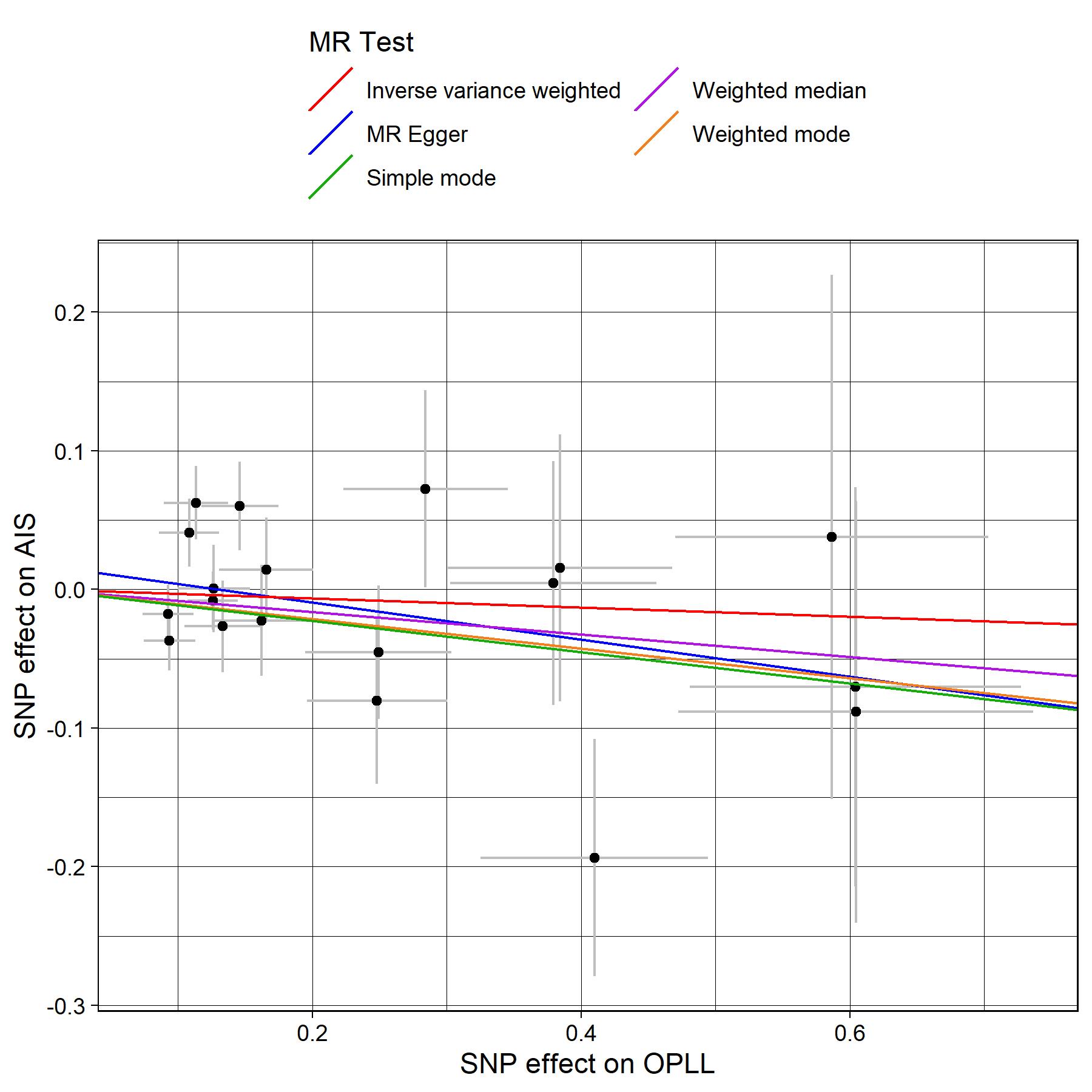

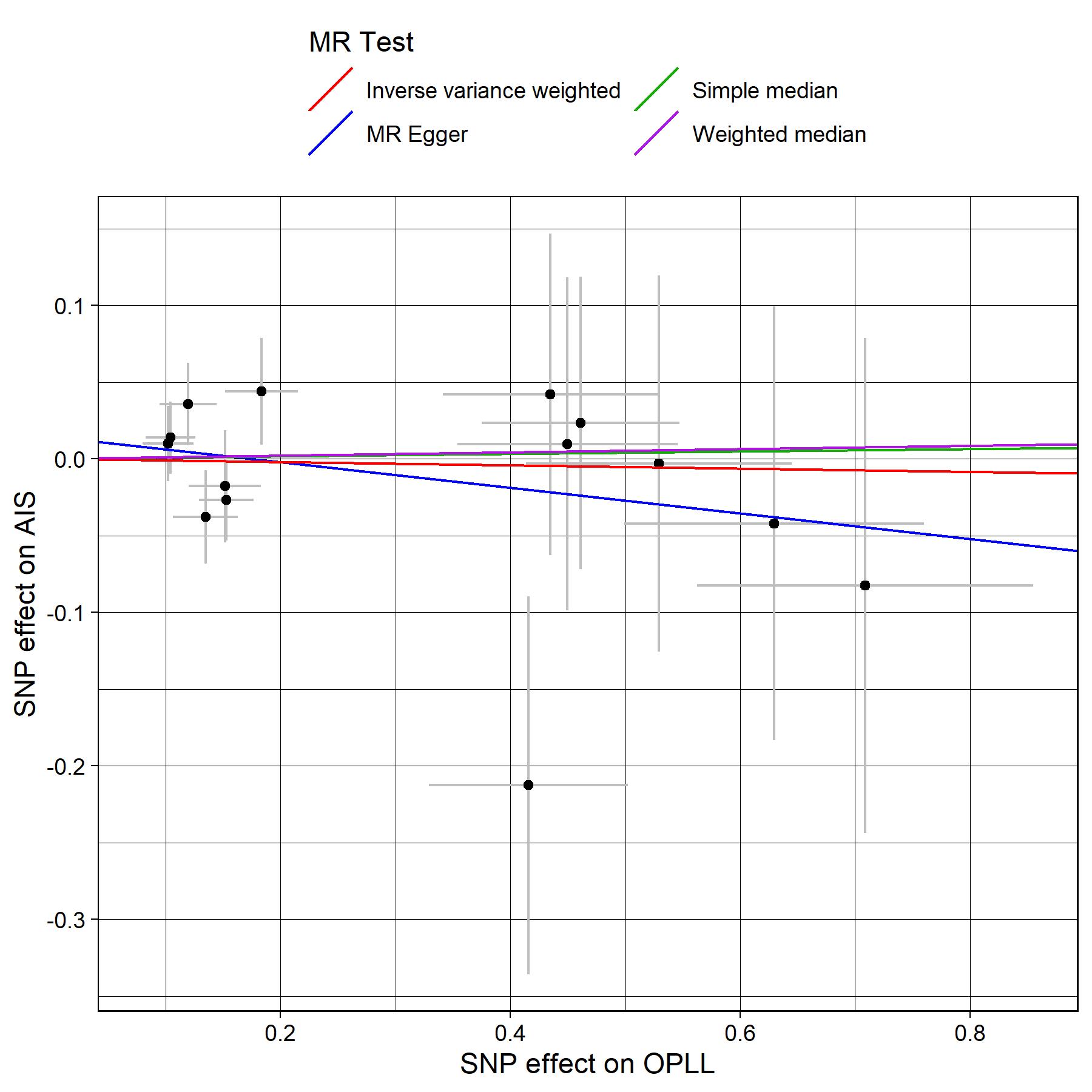
**

**B**

**A**

**
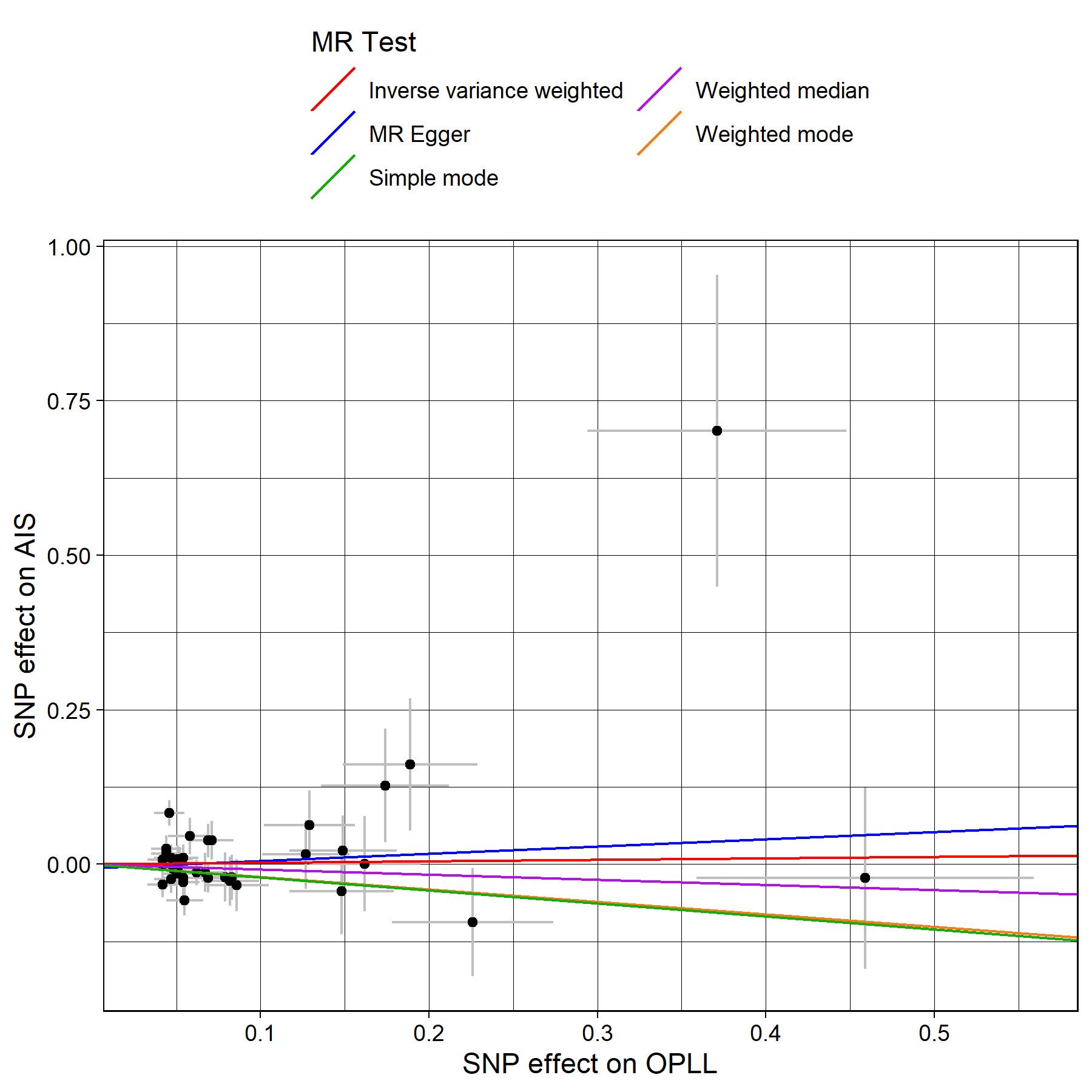
**

SNP effect on AM

**C**

**Supplemental Tables**

**
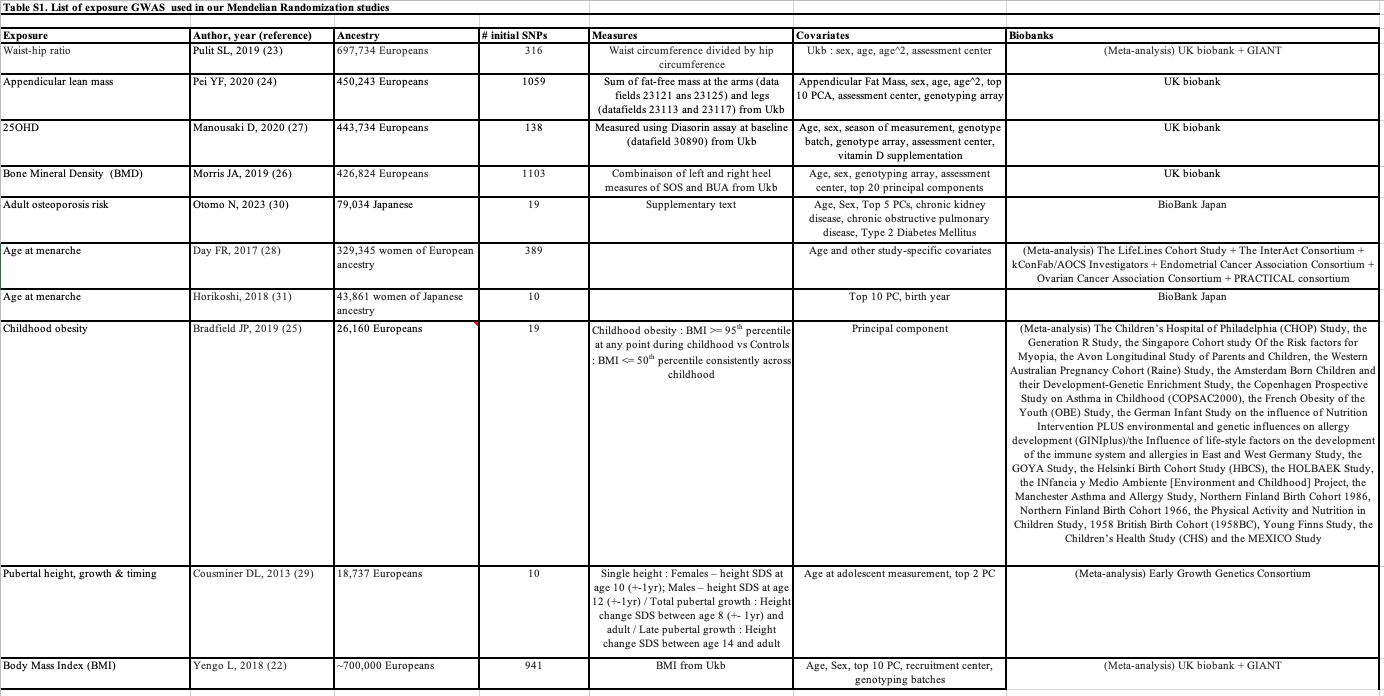
**

**
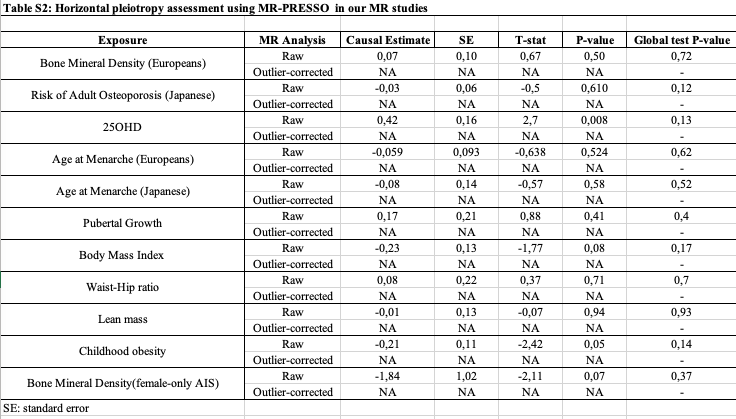
**

**
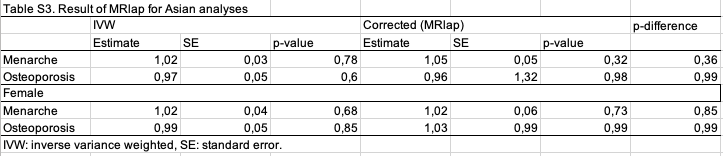
**

**
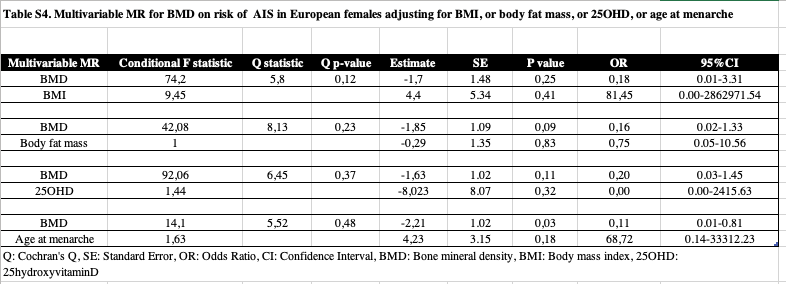
**

**
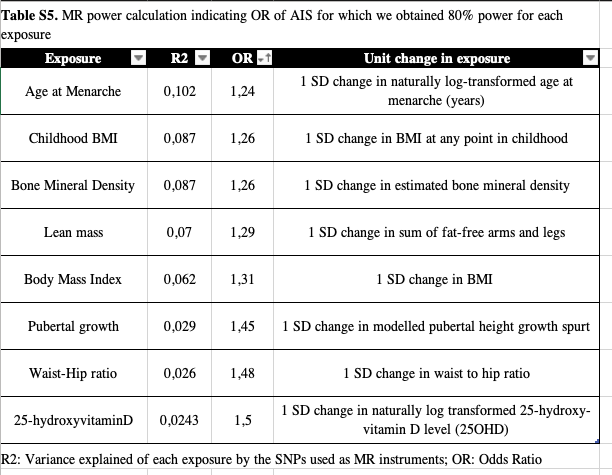
**

**Supplemental references**

1. Nagai A, Hirata M, Kamatani Y, Muto K, Matsuda K, Kiyohara Y, Ninomiya T, Tamakoshi A, Yamagata Z, Mushiroda T, Murakami Y, Yuji K, Furukawa Y, Zembutsu H, Tanaka T, Ohnishi Y, Nakamura Y, BioBank Japan Cooperative Hospital Group, Kubo M. Overview of the BioBank Japan Project: Study design and profile. J. Epidemiol. 2017 Mar;27(3S):S2–8.
2. Hirata M, Kamatani Y, Nagai A, Kiyohara Y, Ninomiya T, Tamakoshi A, Yamagata Z, Kubo M, Muto K, Mushiroda T, Murakami Y, Yuji K, Furukawa Y, Zembutsu H, Tanaka T, Ohnishi Y, Nakamura Y, BioBank Japan Cooperative Hospital Group, Matsuda K. Cross-sectional analysis of BioBank Japan clinical data: A large cohort of 200,000 patients with 47 common diseases. J. Epidemiol. 2017 Mar;27(3S):S9–21.
3. Patterson N, Price AL, Reich D. Population structure and eigenanalysis. PLoS Genet. 2006 Dec;2(12):e190.
4. Akiyama M, Ishigaki K, Sakaue S, Momozawa Y, Horikoshi M, Hirata M, Matsuda K, Ikegawa S, Takahashi A, Kanai M, Suzuki S, Matsui D, Naito M, Yamaji T, Iwasaki M, Sawada N, Tanno K, Sasaki M, Hozawa A, Minegishi N, Wakai K, Tsugane S, Shimizu A, Yamamoto M, Okada Y, Murakami Y, Kubo M, Kamatani Y. Characterizing rare and low-frequency height-associated variants in the Japanese population. Nat. Commun. 2019 Sep 27;10(1):4393.
5. Loh P-R, Danecek P, Palamara PF, Fuchsberger C, A Reshef Y, K Finucane H, Schoenherr S, Forer L, McCarthy S, Abecasis GR, Durbin R, L Price A. Reference-based phasing using the Haplotype Reference Consortium panel. Nat. Genet. 2016 Nov;48(11):1443–8.
6. Das S, Forer L, Schönherr S, Sidore C, Locke AE, Kwong A, Vrieze SI, Chew EY, Levy S, McGue M, Schlessinger D, Stambolian D, Loh P-R, Iacono WG, Swaroop A, Scott LJ, Cucca F, Kronenberg F, Boehnke M, Abecasis GR, Fuchsberger C. Next-generation genotype imputation service and methods. Nat. Genet. 2016 Oct;48(10):1284–7.
7. Zhou W, Nielsen JB, Fritsche LG, Dey R, Gabrielsen ME, Wolford BN, LeFaive J, VandeHaar P, Gagliano SA, Gifford A, Bastarache LA, Wei WQ, Denny JC, Lin M, Hveem K, Kang HM, Abecasis GR, Willer CJ, Lee S. Efficiently controlling for case-control imbalance and sample relatedness in large-scale genetic association studies. Nat Genet. 2018 Sep;50(9):1335-1341.
8. Mounier N, Kutalik Z. Bias correction for inverse variance weighting Mendelian randomization. Genet Epidemiol. 2023 Jun;47(4):314-331.
